# Supplementary figures and images for: PFKFB4 interacts with ICMT and activates RAS/AKT signaling-dependent cell migration in melanoma
Source: Life Sci Alliance. 2022 Aug 1;5(12):e202201377. doi: 10.26508/lsa.202201377 (PMC9348664; doi:10.26508/lsa.202201377)

Figure 1B:

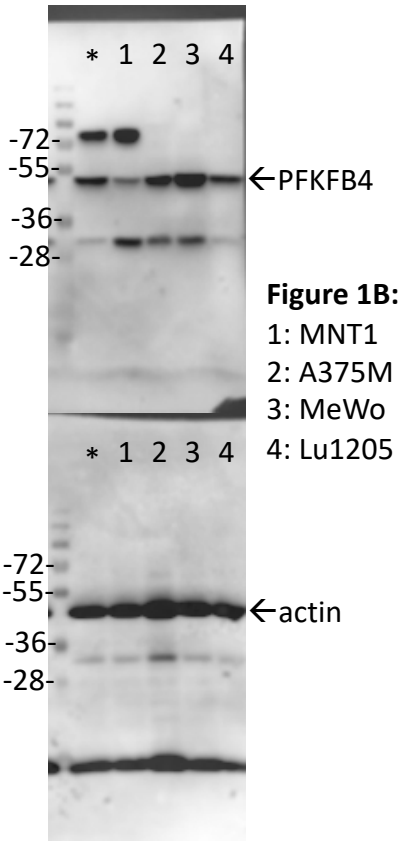

Figure 1C:

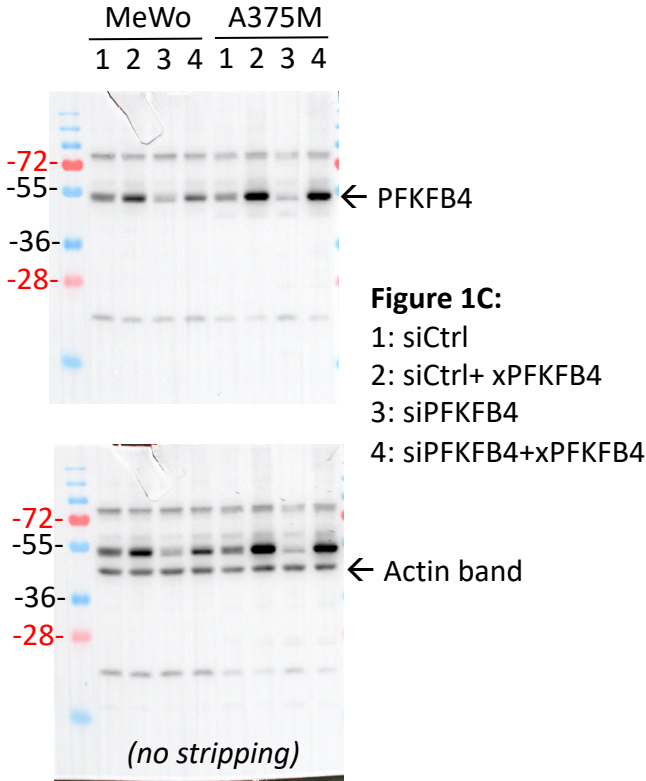

Supplement: Supplementary file 1 [file LSA-2022-01377_SdataF1.1.pdf]

Figure 2B:

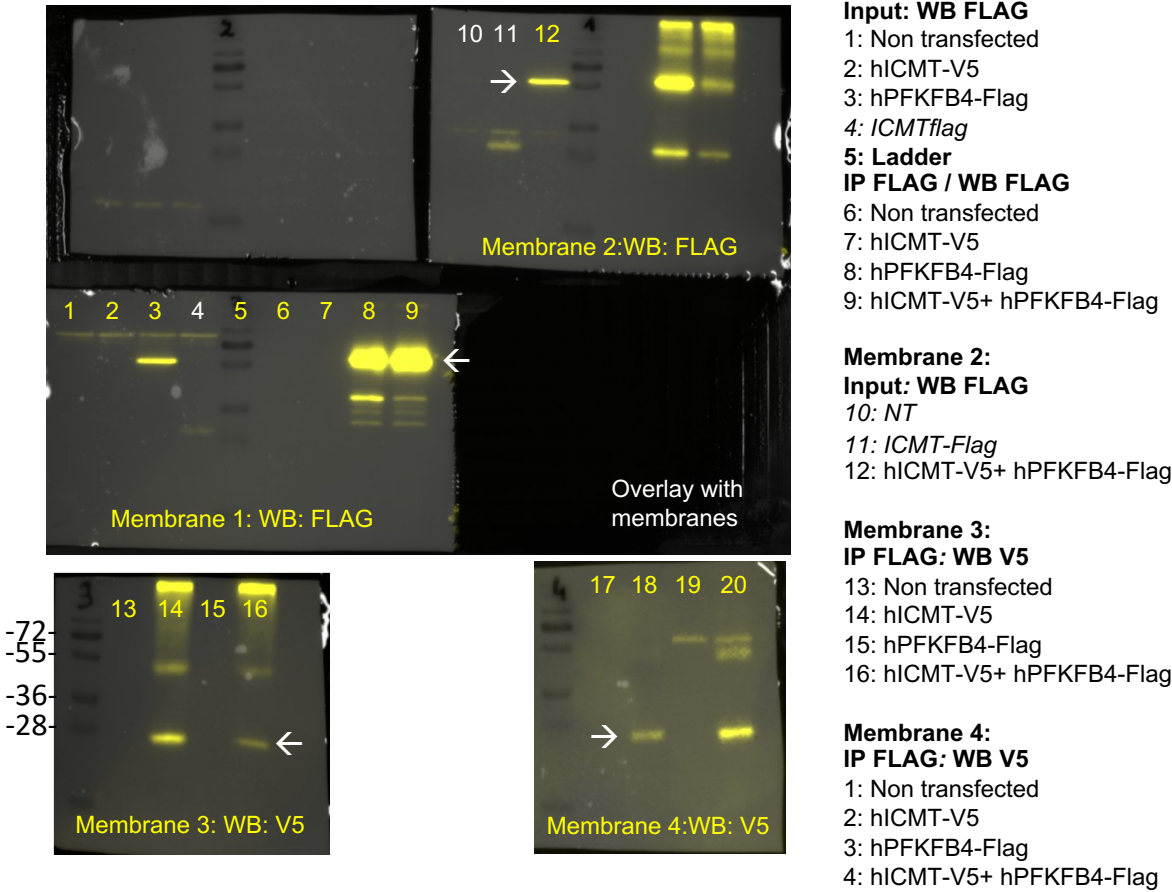

Figure 2D:

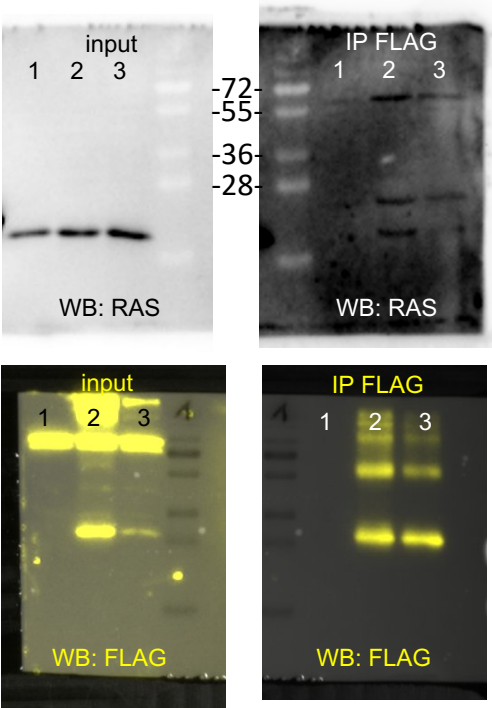

**Figure 2D:**  
1: Non transfected  
2: hCMT-FLAG-Myc  
3: hCMT-FLAG-Myc + siPFKFB4

Supplement: Supplementary file 4 [file LSA-2022-01377_SdataF2.1.pdf]

Figure 4A:

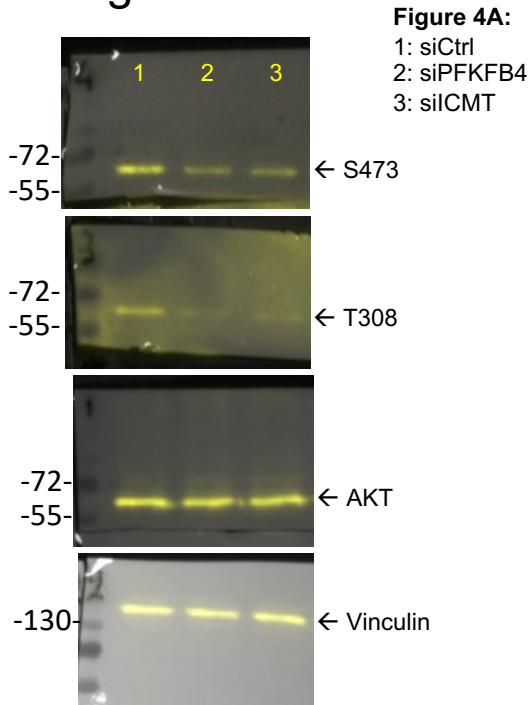

Figure 4C:

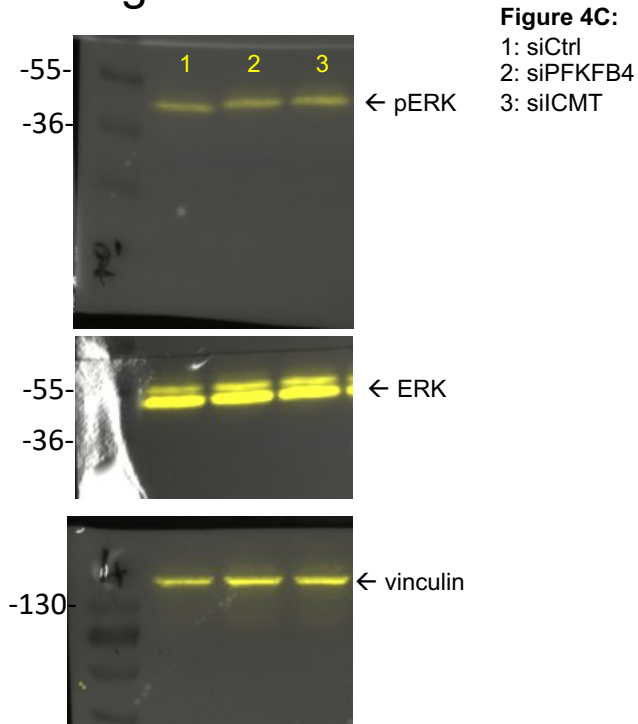

Figure 4D:

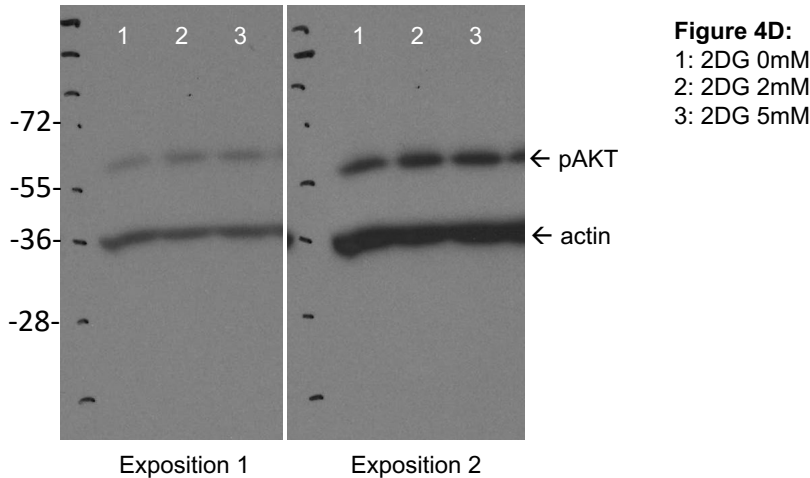

Supplement: Supplementary file 7 [file LSA-2022-01377_SdataF4.1.pdf]
