# Supplementary material for: PFKFB4 interacts with ICMT and activates RAS/AKT signaling-dependent cell migration in melanoma
Source: Life Sci Alliance. 2022 Aug 1;5(12):e202201377. doi: 10.26508/lsa.202201377 (PMC9348664; doi:10.26508/lsa.202201377)
Supplement: Supplementary file 3 [file LSA-2022-01377_TableS1.docx]

**Table S1: small interfering RNAs.**

| siRNA Reference | Sequences | Targeted Exon |
| --- | --- | --- |
| siPFKFB4 (1) Dharmacon Smartpool  D-006764-01/02/04/17 | GAGCGACCATCTTTAATTT | 5 |
|  | CATCGTATATTACCTCATG | 8 |
|  | GAAATGACCTACGAGGAAA | 10 |
|  | GGGACAGGCCTCAGAACGT | 13-14 |
| siPFKFB4 (2) - Invitrogen #HSS107863 | GACCTACGAGGAAATTCAGGATAAT | 10 |
| siICMT- Dharmacon Smartpool  #M-005209-01-0010 | N/A (Court et al., 2017) |  |
| Mouse siPFKFB4 - Dharmacon Smartpool M-054640-01-0010 | CUAAGAAGCUGACGCGGUA  AGAGUCGCAUCGUUUAUUA  CACGGGAACUGACCCAGAA     AUUCAACGUUGGUCAGUAU | 2  8  1  3 |
