# Supplementary material for: PFKFB4 interacts with ICMT and activates RAS/AKT signaling-dependent cell migration in melanoma
Source: Life Sci Alliance. 2022 Aug 1;5(12):e202201377. doi: 10.26508/lsa.202201377 (PMC9348664; doi:10.26508/lsa.202201377)
Supplement: Supplementary file 9 [file LSA-2022-01377_TableS2.docx]

**Table S2: Plasmids**

| Plasmid Name | Reference |
| --- | --- |
| Xenopus pcDNA3-PFKFB4wt-V5 | this work-Gateway cloning |
| Xenopus pcDNA3-PFKFB4-DBM-V5 (G48A;H258A) | this work-Gateway cloning |
| Xenopus pcDNA3-PFKFB4-Nter-V5 (aa1-251) | this work-Gateway cloning |
| Xenopus pcDNA3-PFKFB4-Cter-V5 (aa 251-470) | this work-Gateway cloning |
| Xenopus pCS107-PFKFB4-Flag-HA | this work. |
| Human pcDNA3-HA-Myr-AKT ; « ca-AKT » | Given by Eychene A. |
| Human pcDNA3-p110-caax ; « ca-PI3K » | Given by Eychene A. |
| Human PFKFB4-Flag-myc | Origene RC201573 |
| Human ICMT-Flag-myc | Origene RC207000 |
| Human HRasV12-HA | Given by Eychene A. |
| Human pCS107-ICMT-V5 | This work Cloned from hICMT-Flag-myc |
| MaMTH GAL4 bait and prey destination vectors | Given by Stagljar lab (Saraon *et al*, 2017; Petschnigg *et al*, 2014) |
