# Supplementary material for: PFKFB4 interacts with ICMT and activates RAS/AKT signaling-dependent cell migration in melanoma
Source: Life Sci Alliance. 2022 Aug 1;5(12):e202201377. doi: 10.26508/lsa.202201377 (PMC9348664; doi:10.26508/lsa.202201377)
Supplement: Supplementary file 10 [file LSA-2022-01377_TableS3.docx]

**Table S3: Primers used for Gibson cloning.**

| Final Plasmid | 5'-3' primer sequence |
| --- | --- |
| ICMT-Cub-GAL4 | Fwd1: aaccgtcagaattgatctggtaccaTCGACGTCGACTGGATCCGGTACCG  Rev1: agccaccgccaccCAGGTCCACCTTGACCCCCTTTATG  Fwd2: aaggtggacctgGGTGGCGGTGGCTCTGGAGGTGGTG  Rev2: atcaccgtcatggtctttgtagtctGATTACGTAGAATCGAGACCGAGGAGAGGGTTAGGGATAGG |
| PFKFB4-Nub | Fwd1: tcagaattgatctggtacca*cgcgt*ATGGCGTCCCCACGGGAATT  Rev1: caccgccaccCTGGTGAGCAGGCACCGTGA  Fwd2: tgctcaccagGGTGGCGGTGGCTCTGGAGG  Rev2: cgtcatggtctttgtagtctTTAGATACCTTCCTTGTCTTGAATTTTCGACTTAACGTTGTCG |
| Nub-PFKFB4 | Fwd: ctctggaggtggtgggtccaagcttATGGCGTCCCCACGGGAA  Rev: gggatgccacccgggatcctctagaCTACTGGTGAGCAGGCACC |
