# Supplementary material for: PFKFB4 interacts with ICMT and activates RAS/AKT signaling-dependent cell migration in melanoma
Source: Life Sci Alliance. 2022 Aug 1;5(12):e202201377. doi: 10.26508/lsa.202201377 (PMC9348664; doi:10.26508/lsa.202201377)
Supplement: Supplementary file 11 [file LSA-2022-01377_TableS4.docx]

**Table S4: Primary antibodies.**

| Antibody name /species | Dilution | Provider | Reference |
| --- | --- | --- | --- |
| PFKFB4 Rabbit | 1/2000 | Abcam | ab137785 |
| Phospho-AKT (Thr308) Rabbit | 1/1000 | Cell signaling | 4056 |
| Phospho-AKT (Ser473) Rabbit | 1/1000 | Ozyme | 4060 |
| AKT Rabbit | 1/1000 | Cell signaling | 9272 |
| Actin Rabbit | 1/2000 | Sigma | A2066 |
| Vinculin HVINI Mouse | 1/5000 | Sigma | V9131 |
| Phospho-ERK 1&2 Mouse | 1/1000 | Sigma | M9692 |
| ERK Rabbit | 1/1000 | Santacruz | Sc-93 |
| ICMT Rabbit | 1/1000 | Proteintech | 51001-2-AP |
| HA Rat | 1/1000 for WB;  1/500 for IF | Roche | Clone 3F10 |
| Flag Rabbit | 1/1000 | Sigma | F7425 |
| V5 Mouse | 1/10000 | Abcam | ab27671 |
