# Supplementary material for: PFKFB4 interacts with ICMT and activates RAS/AKT signaling-dependent cell migration in melanoma
Source: Life Sci Alliance. 2022 Aug 1;5(12):e202201377. doi: 10.26508/lsa.202201377 (PMC9348664; doi:10.26508/lsa.202201377)
Supplement: Supplementary file 12 [file LSA-2022-01377_TableS5.docx]

**Table S5: PCR primers.**

| Primers Name | 5'-3' Sequence |
| --- | --- |
| hTBP | Fwd: 5’-CACGAACCACGGCACTGATT-3’  Rev : 5’-TTTTCTTGCTGCCAGTCTGGAC-3’ |
| h18S | Fwd:5’-GTAACCCGTTGAACCCCATT-3’  Rev : 5’-CCATCCAATCGGTAGTAGCG-3’ |
| hpfkfb4 (exon 3-6) | Fwd:5’-TTTTTCTCCCCGACAATGAAGAG-3’  Rev : 5’-CACACAGATGGACRCGACAAA-3’ |
